# Coordinating Healthcare and Long-term Care: A Four-Country Comparison

**DOI:** 10.34172/ijhpm.9130

**Published:** 2026-07-12

**Authors:** Claus Wendt, Eva Willis

**Affiliations:** ^1^Chair of Sociology of Health and Healthcare Systems, University of Siegen, Siegen, Germany.; ^2^Sociology of Health and Healthcare Systems, University of Siegen, Siegen, Germany.

**Keywords:** Coordination, Healthcare, Long-term Care, Digitalization, Comparison, Typologies

## Abstract

**Background:**

This comparative study examines responsibilities for coordination of healthcare and long-term care (LTC) in Germany, the Netherlands, Sweden, and Switzerland. As populations age, frailty and multimorbidity are increasing, leading to greater care needs and coordination of care between sectors. We analyze how coordination responsibilities among healthcare professionals and organizations vary between different healthcare and LTC systems and what strategies are used for better coordination.

**Methods:**

Fifty qualitative expert interviews were conducted with participants from local government agencies, social health insurances, various healthcare organizations, and professionals directly involved in healthcare and LTC provision. Data were analyzed with qualitative content analysis. The comparative research is based on existing healthcare and LTC typologies.

**Results:**

This study shows the need for clear lines of responsibilities and financial allocation to minimize fragmentation of care coordination. Sweden and the Netherlands assign formal coordination roles to general practitioners (GPs), district nurses, and municipal services. Germany’s approach heavily relies on the family, and in Switzerland, public and private LTC providers are often responsible for coordination. Digital technologies are increasingly used for communication and coordination. In Germany and Switzerland, where GPs have no gatekeeping role and primary healthcare centers are less developed, older GPs are unwilling to invest in digitalization. In Sweden and the Netherlands, where GPs coordinate primary healthcare and referrals to specialists, there is a strong interest in digitalization to improve communication and coordination. Still, digital systems for healthcare and LTC are not fully harmonized.

**Conclusion:**

The comparative study shows the need for clear responsibilities, systemic support, and financial recognition of care coordination to facilitate patient-centered care coordination. Digital technologies and in particularly integrated electronic records across sectors are reported as important tools that can facilitate care coordination across healthcare and LTC.

## Introduction

**Key Messages**

**Implications for policy makers**

- Typologies help to identify countries with similar challenges and solutions for improving coordination of healthcare and long-term care (LTC).
- Interviews in Germany, Netherlands, Sweden, and Switzerland show the importance of clear responsibilities to better connect healthcare and LTC.
- Hospital discharge teams, general practitioners (GPs), district nurses, primary healthcare centers, and local government are key for coordinating patient transfers across healthcare and LTC sectors.
- Model projects suggest that integrating electronic records across sectors can support communication and coordination across healthcare and LTC.

**Implications for the public**

**Less fragmentation:** Moving from an open house model with unconnected providers to a streamlined, team-based approach.

**Smoother hospital-to-home transitions:** Reducing risk of being discharged without a clear care plan and avoidable readmissions.

**Clear go-to contact:** One responsible professional (like GP, district nurse, case manager) to coordinate care and simplify communication.

**Seamless information sharing:** Enabling secure, shared digital records so that patients do not have to repeat their stories and providers are informed.

Patients are often discharged from hospital care once acute medical treatment ends before all care needs are managed or they can independently handle daily activities, raising early readmission risk. Interviews in all countries show the importance of clear responsibilities to better connect healthcare and long-term care (LTC). Assigning a responsible actor such as general practitioners (GPs), district nurses, primary healthcare centers, or municipalities, together with better-integrated electronic records across sectors, may help streamline service coordination for patients and their families.

This article analyzes coordination of healthcare and long-term care (LTC) services in Germany, Netherlands, Sweden, and Switzerland. In modern welfare states, due to demographic changes, an increasing number of people need high-quality healthcare.^1^ To handle these pressures, one potential solution is to enable close cooperation of service providers and coordination of services between healthcare and LTC.^2^ Coordination could prevent poor quality of care, discontinuities in the care process, high admission rates, and overburden of patients and their families.

 Studies have found that individual, organizational, and institutional aspects improve coordination of care, namely different forms of coordination models, intensified forms of communication, clear coordination responsibilities, and monetary recognition of coordination work. Beyond a structural process, a relational framework conceptualizes networks of communication of those tied in the work process of coordination.^3^ Successful coordination, then, stems from a combination of structural, relational, interpersonal, and normative integration.^4^ Different types of coordination are preferred in different sectors. While hospitals tend to favor disease-specific integrated care pathways, primary care tends to prefer patient-centered models due to their flexibility. Communication may be intensified in various ways with general practitioners (GPs) expressing the need for informal dialogue with other primary care professionals.^5,6^ Cross-sectoral access to patient records is crucial for good coordination.^7^ Working culture around care coordination can be improved through reciprocating knowledge of everyday work across sectors by spending time in each other’s workspace and through informal meetings.^8^ A major issue in improving coordination is that specific care coordination responsibilities between sectors rarely exist and that coordination is not recognized organizationally or financially.^6,7,9^ In Germany, GPs are skeptical about an external navigation role.^9^ In Sweden, transfer of older patients from hospital to homecare is mainly coordinated by nurses and discharge teams.^7,9^

 Going beyond earlier comparative studies in this field, we seek to understand how coordination is related to the institutional framework of different healthcare and LTC systems. These systems are based on different modes of financing, regulation, and service provision. We refer to typologies of healthcare and LTC systems to identify their country-specific institutional logic.^10-13^ The mix of healthcare- and long-term types represented by the selected countries—Germany, Netherlands, Sweden, and Switzerland—offer a unique constellation for analysis.

 Our main research question is how and through which processes institutional settings support different concepts of coordination in healthcare and LTC. We also ask who, according to actors and organizations in this field, should be responsible for coordination. We explore whether and how stronger involvement of state actors contributes to coordination of healthcare and LTC and to the explicit assignment of coordination responsibilities.

 Past experience has shown that state, private, and social actors have achieved yet also failed to achieve certain health policy goals.^14^ This has been demonstrated in studies on cost containment,^15-17^ availability of healthcare providers and access to care,^14,18,19^ and quality of services.^20,21^ Based on the interviews, our objective is to analyze how institutional logics shape the assignment of coordination responsibilities and perceptions of successful coordination across healthcare and LTC.

 The next section provides information on methods used for data collection and data analysis. The third section describes institutional characteristics of healthcare and LTC systems under study. The result section four analyzes the role of actors in the coordination process by considering the institutional context. In the final section we discuss the advantages of combining an institutional analysis with the analysis of expert interviews for understanding coordination processes.

## Methods

 This study examines care coordination, namely transition from inpatient hospital care to LTC and integration of medical, nursing, and social care at home. For the analysis, three levels of coordination are defined which seem entangled in everyday practice: system, organizational, and professional.^22,23^ The system level defines the scope, boundaries, and regulations of formal services, affecting access and financing. Formalization and service allocation vary across countries, with public financing affecting supply and demand, linking system and organizational levels. The organizational level shapes service delivery based on system rules, digitalization, and financing. The professional level focuses on service delivery, shaped by hierarchical or horizontal division of labor. Professionalization impacts service provision, with the role of medical doctors and allied health professionals varying across countries. Nurse academization reflects the professional capacity and readiness of the largest allied health workforce group to assume complex coordination roles, a key consideration in light of the overwork of GPs across all systems. Coordination is shaped by actors’ perceptions, collective culture, tools like electronic patient records, and national contexts.^2^ First, we describe healthcare and LTC typologies, based on quantitative methods that were used to analyze the system level of healthcare and LTC, followed by qualitative methods detailing expert interviews selection, conduct, and analysis.

###  Healthcare and Long-term Care Typologies

 Typologies outline key characteristics of healthcare and LTC systems and allow to better understand welfare-state similarities and differences, institutional characteristics, actors, and coordination processes, including impact on satisfaction and health inequalities.^24-32^ We use two existing typologies: the healthcare system typology by Reibling et al^11^ and the LTC typology by Ariaans et al.^13^ Both frameworks use dimensions such as supply, public-private mix, access regulation, and performance, enabling comparative analysis across sectors. Typologies gained prominence in the 1990s and belong to a broader scholarly tradition of constructing healthcare system models based on dimensions like expenditure, financing mix, out-of-pocket payments, resource availability, and access.^10,27,33-35^

 Various clustering techniques exist to classify countries for healthcare and LTC typologies.^10,12,33-36^ Reibling et al^11^ and Aarians et al^13^ build types and grouping of countries on many different cluster analyses (both hierarchical and k-means), with 24 cluster results used in both studies. This approach identifies both strong as well as moderate links between countries. Reibling et al^11^ classify 29 Organisation for Economic Co-operation and Development (OECD) countries into five types of healthcare systems. Ariaans et al^13^ classify 25 OECD into six LTC types. Both studies included OECD healthcare data, further international datasets and Healthcare System in Transition series. Values were double-checked by contacting country experts. The countries that are studied in this paper represent four of five healthcare system types by Reibling et al^11^ and three of six LTC types by Ariaans et al.^13^

 We selected cases to span the major institutional logics (public–private regulation, gatekeeping vs. free access, integrated vs. fragmented LTC), not to cover all possible types. Two of the selected countries, Sweden and the Netherlands, are among the leading countries in digitalization and in coordinating healthcare and LTC. Other countries with less digitalization, such as Germany, may benefit from digitalization and coordination paths. Including all typological variants would have diluted depth and comparability of the expert material but may be included in future research. We evaluate how well the typologies travel across institutional realities by applying rather than reconstructing typologies.

###  Qualitative Methods

 To analyze coordination processes, we conducted interviews with organizational experts responsible for financing, organizing, or delivering care. Participants included local governments, healthcare and LTC insurance companies, non- and for-profit care service providers, hospitals, and various professions (See Table 1). Purposive sampling, including supplementary snowball sampling, ensured a diverse perspectives within each country.^37-41^ The specific organizations interviewed varied by country, as some types were either non-existent or not relevant to coordination in a given context. However, in each country, relevant stakeholders such as client and consumer organizations were included when identified.

**Table 1:** Interviews by Country and Category

	**Germany**	**Netherlands**	**Sweden**	**Switzerland**
GPs	2	3	-	1
LTC nurses	2	2	2	1
Transfer nurses	1	1	1	-
Geriatrician	1	2	-	-
Hospital	1	-	-	1
(Home)care provider	3	1	4	2
Funding agencies	1	2	-	1
Supervisory agencies	-	1	3	1
Care assessment	1	1	-	-
Other (eg, clients)	5	2	-	1
Total	17	15	10	8

Abbreviations: LTC, long-term care; GPs, general practitioners. Source: Bahle et al.^2^

 Fifty interviews were conducted in 2019 and 2020: 17 in Germany, 15 in the Netherlands, 10 in Sweden, and 8 in Switzerland. Interviews lasted 40 to 60 minutes with a pre-tested semi-structured interview guide,^2^ considering national specificities and current reforms. The interview guide covers: (*a*) recent developments in healthcare and LTC, (*b*) transition from hospital to homecare, (*c*) provision of integrated healthcare and LTC at home, (*d*) rehabilitation and prevention, and (*e*) electronic tools for coordination. Interviews in Germany and Switzerland were conducted in German and interviews in Sweden and the Netherlands were held in English with some interviews including multiple experts or translators. Interviews were audio-recorded with consent of interviewees, transcribed, and pseudonymized.^42^ Interviews are numbered with a country code and an acronym for the organization (eg, NL1-CIZ; see list of organizations quoted in this study in Supplementary file 1). According to the University Siegen’s regulations, the study did not require formal ethics committee approval because no patient data or vulnerable populations were involved. All interviewees provided informed consent and interviews were pseudonymized.

 We applied qualitative content analysis to analyze the data.^40,43^ English transcripts of the interviews were coded using MAXQDA 2020 with a deductive coding scheme that was tested and modified before all interviews were coded, supplemented by inductive elements.^40,42^ At least one interview per country was coded independently by two researchers. The coding scheme distinguishes between “interface” (transition from hospital to homecare, homecare setting, rehabilitation, electronic tools) and “integration level” (system, organizational, professional). “Interface” refers to the location of coordination. The category “responsibility” was developed with codes for the responsible actor (eg, patient, GP, primary care center, homecare provider, hospital transfer nurse, district nurse, government, municipality, social insurance).^2^

## Results

 Typologies identify institutional logics that shape how coordination mechanisms operate. The subsequent sections triangulate these structural characteristics with expert interview data, illustrating how financing, local governance, professional relations, and digital infrastructure within different institutional combinations. This integration allows us to connect cross-national system design with on-the-ground coordination practices. Typologies differ in areas that are more specific to the respective field of social policy.^44^ For example, primary care orientation is less relevant for LTC than for healthcare, and cash benefits are not relevant anymore for healthcare systems but still for LTC. The four countries represent different combinations of healthcare and LTC typologies (Table 2).^11,13^

**Table 2:** Healthcare- and Long-term Care Types

**Country**	**Healthcare System Type** ^11^	**Long-term Care Type** ^13^
Germany	Supply-and choice-oriented public system	Private supply systems
Netherlands	Regulation-oriented public systems	Private need-based supply system
Sweden	Performance-oriented public systems	Public supply system
Switzerland	Supply- and performance-oriented private systems	Private need-based supply system

Source: Own depiction.

###  Typology-Based Institutional Context

 The healthcare system and long-term system typologies are contextualized with qualitative expert interviews who identified four institutional differences that influence coordination in healthcare and LTC. First, the division of primary, secondary, and LTC sectors, including financing processes. The degree of institutional and financial segregation between secondary healthcare and LTC influences coordination of care. Second, the responsibility of local government. Alongside national government, local governments provide legal and structural conditions for coordination. In many countries, municipalities are involved in coordinating, financing, and providing healthcare and LTC services. Third, what roles professions take in coordination and how this is entangled with the scope of their practice. GPs play a crucial role in coordination as the first point of contact and primary actor in transferring patients between sectors. In some countries, GPs have a gatekeeping role, meaning patients are registered with one primary care facility where GPs coordinate care, directing referrals to and from specialists and inpatient hospitals. Fourth, how the level of digitalization is integrated in the sectors and utilized for coordination processes.

 The effectiveness of digital tools in care coordination is often contingent on pre-existing structural roles, such as GP gatekeeping, which provides a natural hub for information exchange. Information sharing is more transparent as patients are registered with primary care facilities that act as a single point of contact. Conversely, digitalization strengthens this traditional model by making the gatekeeper’s coordinating role more efficient, demonstrating an interdependence between institutional design and technological advancement. This logic extends to newer structural innovations, such as assigning coordination responsibilities to allied health professionals, and countries without gatekeeping roles, where digital systems can similarly empower these roles by streamlining communication and data access. In such institutional contexts, digitalization is used to support exchange of information among healthcare providers. After the end of the country analysis we present a synthesis of findings and recommendations that interviewees made to improve coordination.

###  Coordination in Public-Public Systems (Sweden)

 Sweden represents in healthcare a primary care oriented public type and in LTC a public supply type. Sweden features a strong and direct public influence over healthcare and LTC, with gatekeeping and limited patient choice.^11^ In this context, GPs and primary care centers are responsible for coordinating the process between primary healthcare and hospital care^45^ and are a candidate for coordinating healthcare and LTC. Nurses are also potential coordinators, particularly nurse practitioners and advanced nurse practitioners, as master’s level educated autonomous practitioners. This model is in an early development phase and nurse practitioners could take over responsibility of coordinating complex care needs.^46,47^ The large number of nurse assistants and the comparatively low number of nurses with higher education in Sweden might limit the coordinating role of the nursing profession.^2^ As a digitalization leader, Sweden can leverage eHealth technologies, though an integrated electronic system for both healthcare and LTC is still missing.^48^

####  Division of Primary and Secondary Healthcare and Long-term Care 

 In Sweden, primary and secondary healthcare and LTC are highly integrated, although there is an institutional and financial separation with hospitals being the responsibility of counties, and LTC the responsibility of municipalities. Both government levels have high degrees of autonomy. Various laws and procedures have been implemented to strengthen care coordination and collaboration between healthcare and LTC, such as individual care plans. If patients stay in hospitals longer than medically necessary because LTC is not provided in time, municipalities have to compensate hospitals for this period. Such rules create a financial incentive for municipalities to ensure continuity of care. If the municipality:

 “*cannot take the patient home, they have been forced to pay the inpatient stay. So, they tried to create places to take them home”* (SE6-SBU).

 Coordination responsibilities require additional resources as municipalities have come under financial pressure since the need for LTC and rehabilitation has increased for patients who are discharged from hospital earlier (SE3-IVO; SE7-Socialstyrelsen).

####  Local Government 

 In Sweden, municipalities have a strong financial incentive to organize homecare after hospital discharge. Delayed hospital discharges are seen as a sign of coordination shortcoming at the regional or municipal level.^49^ District nurses, employed by counties or municipalities, play a key role in coordination. They serve as permanent contacts for patients and, if needed, receive support from mobile doctors. Local or county-level model projects in Sweden aim to integrate healthcare and LTC even more. Interviewed organizations state that municipalities consider wishes and needs of older people when organizing LTC. Model projects with a higher level of coordination of elderly care are rated very positively.

####  Role of Professions

 Sweden has a functional gatekeeping system that lacks a strong regulatory framework. Primary care is prioritized and organized in primary care centers where GPs, nurses, therapists, midwives, and psychologists cooperate.^50,51^ About 40% of these centers are privately-, and 60% county-owned, with predominantly salaried physicians.^50^ Organizations complain that actors from primary care centers, in particular GPs, are hardly involved in homecare.

 “*They’re all so booked up with their patients at the healthcare center. So, we [district nurses] don’t have much assistance”* (SE8-SSF).

 Interviewed organizations call for GPs to cooperate to a higher extent with district nurses employed by county or municipality.^52^ Collaboration of district nurses varies based on their employment. County-employed district nurses collaborate more with GPs, while municipality-employed district nurses work with homecare providers and therapists. In the latter case, they lack contact with GPs and access to patient information.^53,54^ Such responsibilities can be fulfilled by academically trained nurses but not by semi-skilled personal carers or nurse assistants that:

 “*understands different rules, different organizations” *(SE10-Tryggt mottagande).

 In homecare, organizations regard collaboration between GPs and district nurses as crucial. Both professions coordinate hospital transitions and the work of homecare providers such as occupational therapists and physiotherapists.

####  Digitalization

 In Sweden, digitalization of healthcare and LTC is highly advanced. The Swedish eHealth Agency in 2014 signaled digital healthcare as a national priority.^55^ By 2018, nationwide patient-accessible electronic health records were introduced und telemedicine has since grown.^56^ eHealth policy focuses on integration and interoperability across sectors and regions.^55^ Except for model projects, the electronic system for homecare is still separate from electronic patient records.^57^ Organizations view this as a major deficit, particularly for LTC staff who lack access to the medical database. Healthcare and LTC providers spend considerable time collecting relevant information which reduces time for service provision. Not all necessary information is provided in time with a negative effect for safe and quality care. Organizations criticize the lack of a nationwide medication database that is accessible for healthcare and LTC providers. They agree that full implementation of a national system would enhance care coordination, information provision, and mutual planning and service provision by healthcare and LTC professions.

 “*The dream would be that for example the nurse comes … to the patient with the laptop and has a direct discussion with the patient’s doctor or a social worker and solves the problem right away” *(SE5-Regional council).

 Data protection is seen as essential, but organizations agree that it should not hinder exchange of information needed for high quality services (SE9-Tiohundra).

###  Coordination in Need-Based Systems (Netherland)

 Netherlands represents a regulation-oriented public type in healthcare and a need-based supply type in LTC. Some scholars still consider the healthcare system of the Netherlands as private.^58^ Yet, the healthcare system is characterized by even stronger state regulation than Sweden’s.^10,11,59^ GPs and primary care centers manage access through a strong gatekeeping role. The Netherlands started academization of nurses very early,^60^ resulting in many nurses with higher education.^61^ The country is also at the forefront of digitalization and has started processes that could enable communication through digital tools between healthcare and LTC providers.^2^

####  Division of Primary and Secondary Healthcare and Long-term Care

 The Netherlands has a highly integrated system. As homecare has become the financial responsibility of health insurance, this reinforces allocation of responsibilities for medical, nursing, and social care. Healthcare, residential LTC, homecare, and home help are in the responsibility of different organizations at national, regional, and municipal levels (NL7-NHG; NL15-ZN). According to interviewed organizations, financing structures in the Netherlands, like in the other countries, lead to cost shifting (NL7-NHG; NL13-Verenso). Access to residential care has been severely restricted through the 2015 reform and municipalities have become more influential in non-24-hour care and for assistance to the elderly. When organizational and financial borders are lower, for instance when hospitals and homecare are in the same ownership or when competitors are lacking, coordination is improved (NL7-NHG). In competitive areas where GPs face numerous unknown providers, communication is difficult. In contrast, being the sole homecare provider fosters regular contact and trust with all GPs, enabling them to “easily pick up the phone and call” (NL4-homecare nurse). The number of competing homecare organizations is considered to be too high (NL12-V&VN; NL15-ZN). Interview partners criticized market oversaturation, noting that numerous competing homecare providers in one city hinder coordination. They argued for centralized cooperation through a shared contact point, contrasting this with the complexity introduced by marketization (NL1-Actiz).

####  Local Government 

 In the Netherlands, municipalities are responsible for social care under the 2015 Social Support Act. The reform restricted access to nursing homes, making elderly with lower care needs ineligible for publicly funded stationary LTC.^62^ Health insurances are responsible for community nursing and body-related personal care, which strengthens local healthcare and LTC collaboration. Municipalities are major actors with financial allocation powers as they manage all other non-residential care services. Needs assessment centers (CIZ) assess residential care recipients, while district nurses assess eligibility for home nursing, and municipalities assess home assistance. Overlapping services negatively impact continuity of care between different schemes. Some organizations call for municipalities to take a larger role in coordination (NL2-CIZ). Financial uncertainty in unclear cases, such as mealtime guidance, leads to disputes over payment. This negotiation resulted in a split-financing solution where insurers cover the first two days, after which municipalities are required to assume responsibility and make permanent arrangements (NL15-ZN).

####  Role of Professions

 Gatekeeping of access to secondary and LTC is part of the family doctor model in the Netherlands. During transition from hospital to homecare, organizations suggest various professional groups for coordinating tasks: homecare nurses (NL9-Patientenfederatie), transfer nurses as central actors supported by homecare nurses, GPs, and geriatricians (NL10-Transfer nurse). Some organizations suggest shared responsibilities by GPs, transfer nurses, geriatricians, and community nurses (NL1-Actiz), and by homecare nurses and transfer nurses (NL14-VGZ). GP’s are not seen as central actors for coordinating transition from hospital to homecare alone (NL13-Verenso). Building on concerns about GP overwork, interviewees questioned whether coordinating non-medical care tasks should fall within a GP’s remit (NL7-NHG).

 More shared responsibilities are sought in the transition from hospital to homecare. Most organizations consider GPs to be responsible for coordination, either alone (NL13-Verenso), supported by a community nurse (NL8-NZa; NL12-V&VN), in a team including homecare nurses (NL1-Actiz; NL5-Huisarts; NL14-VGZ), or in a team with homecare nurses and social workers (NL6-Ineen). Some organizations also see homecare nurses in the main coordinating position (NL4-Homecare nurse), or as main coordinator together with GPs (NL9-Patientenfederatie). At the system and organizational level, organizations call for municipalities to play a greater role in coordinating homecare, supported by cross-sector compatible electronic patient records (NL2-CIZ; NL6-Ineen; NL8-NZa; NL9-Patientenfederatie; NL10-Transfer nurse; NL15-ZN).

####  Digitalization

 The Netherlands belongs to the leaders in healthcare and LTC digitalization. All organizations in both sectors use electronic systems. Interviewees described electronic records, video calls, and pharmaceuticals dispensed by electronic devices as supporting service provision and coordination across sectors and professions (NL6-Ineen; NL14-VGZ; NL15-ZN). Organizations suggest further improvement of information sharing that would require a compatible electronic system in healthcare and LTC. Currently, they are not compatible, and patients and their families often transfer information between sectors (NL3-Geriatrician; NL4-Homecare nurse; NL7-NHG). Practitioners call for access to an integrated platform of all sectors (NL3-Geriatrician), as non-compatibility creates an additional time-burden:

 “*with mailing, telephoning, logging into systems … you have a password for this, a password for that”* (NL 10-Transfer nurse).

 First model projects aim to harmonize electronic communication systems for healthcare and LTC (NL7-NHG). Dutch organizations consider data protection important, but privacy issues should not delay implementation of electronic records and other forms of communication and coordination (NL3-Geriatrician; NL15-ZN).

###  Coordination in Public-Private Systems (Germany)

 Germany represents a public supply and choice-system in healthcare and a private supply system in LTC. The importance of choice and the low level of access regulation suggest that coordination is much needed but remains fragmented with many coordination responsibilities remaining with family members.^2^ As LTC is provided by many competing public and private providers, coordination is more important than in systems with few or a single provider. Germany, a latecomer in digitalization and nurse academization, may therefore face greater coordination challenges than Sweden and the Netherlands.

 Coordination is dependent on institutional factors, such as waiting for confirmation of funding for rehabilitation or LTC. Hospitals primarily manage discharge during the transition to homecare, with varying quality. Larger hospitals are usually better equipped to coordinate discharge. Interviewees suggest greater involvement of homecare providers and social workers. This is crucial since hospitals’ responsibilities end at discharge, while patients need ongoing support by homecare and LTC services.

####  Division of Primary and Secondary Healthcare and Long-term Care

 Germany has a strict separation between financing of healthcare and LTC. The healthcare system is organizationally and financially further divided into inpatient and outpatient care. Separate financing means that hospitals provide some coordination of care but the focus remains on discharge and not continuity of care. Other countries may have services such as hospital-at-home that operate in the community but are hospital-based to either expedite discharges from hospital or avoid hospital admissions. They are therefore coordinating care outside hospitals. Coordination of the fragmented LTC system is mostly organized by next of kin. Interviewed organizations identified three institutional problems for care coordination: diagnosis related group (DRG) system leading to early hospital discharges and increased pressure on discharge processes, insufficient post-discharge care capacities, and too much bureaucracy. As described by a discharge manager:

 “*My workload per patient has significantly increased. Because it is not enough to contact ten nursing homes, but thirty, forty, fifty, and you also need to expand the search radius”* (DE7-Discharge management).

 Patients are often discharged without adequate post-discharge care. Shortages of short-term care, rehabilitation, and nursing homes exacerbate these issues. Responsibilities in care coordination often remain in the hands of relatives. High levels of bureaucracy further delay care coordination. Strict rules and lengthy application processes for level-of-care assessments complicate planning transitions from hospital to homecare, as funding is uncertain and coordination is not monetarily recognized. Because homecare cannot commence until payment is approved, some patients are left discharged without guaranteed care for days, which is experienced as unacceptable (DE5-Care support center).

####  Local Government 

 In Germany, municipalities are hardly involved in healthcare and LTC. They run and finance some LTC homes but are in general not involved in homecare.^63^ Municipalities are responsible as a last safety net^64^ and, according to interviewees, should engage more in connecting local structures and providers. Organizations suggest that municipalities should receive additional resources and employ district nurses.

####  Role of Professions

 Germany has no gatekeeping and GPs are not seen as main coordinators. Interviewed organizations consider GPs, homecare providers, and social workers as potential coordinators for homecare, though opinions vary on GPs’ roles. Several organizations argue that GPs should be key coordinators, as they recognize early symptoms of deterioration, organize specialist examinations, and communicate with relatives and homecare staff. Still, many criticize staff shortage resulting in high workload of GPs who do not have capacities for coordination (DE15-KBV).

 Building on concerns about GP capacity, the National Association of Statutory Health Insurance Funds suggest coordination to be performed by other professions. GPs should not be overburdened, as care coordination does not require a physician (DE13-GKV).

 Few nurses have a higher education background with few organizations in Germany supporting an academization of nursing to converge competences of GPs and nurses. Expertise of medical doctors is primarily seen in medical tasks and coordination of LTC in the responsibility of nurses.

 “*Tasks in which nurses are clearly more competent than physicians should be performed by nurses.... But in Germany, there is a very strong doctor’s prerogative for all activities”* (DE1-BAGFW).

 In LTC too many actors are involved in tasks such as general care, wound care, physiotherapy, and social care. Patients in specialized outpatient care frequently describe an overwhelming open house day scenario. This arises from a fragmented model where a parade of different providers visits, each responsible only for a single, specialized task such as basic care, tube feeding, or wound management (DE4-BV Geriatrie).

####  Digitalization

 In Germany, electronic patient records are in an early stage. Interviewed organizations echo the hope that electronic tools have a great potential to improve information sharing and interprofessional collaboration to:

 “*get people around the table more quickly in order to work together on a case”* (DE11-DV).

 In addition to better service coordination, they expect digital tools to save time on documentation, provide better information on healthcare and LTC, and improve access through video consultations in rural areas.

 Organizations agree that digital tools cannot replace personal contacts and services from healthcare and LTC professionals but can support information exchange and coordination. They criticize that the nationwide electronic record is not fully implemented and provides information mainly to patients rather than providers, is limited to healthcare, and does not include LTC. Almost all organizations emphasize the importance of data protection. They also highlight problems that may lead to lower acceptance of digital tools in Germany, such as high costs, high number of older GPs, and concern that healthcare and LTC professionals lose autonomy under the control of digital systems.

###  Coordination in Private-Need-Based Systems (Switzerland) 

 Switzerland is a supply-oriented private healthcare system and like Netherlands a need-based supply system in LTC. Regulation is low and patients generally have free choice of doctors. Academization of nurses and digitalization in healthcare and LTC are both at a low level, with possible restrictions for coordination.

####  Division of Primary and Secondary Healthcare and Long-term Care

 In Switzerland, hospitals hold little control over the provision of care outside secondary care. While interviewees report that the financing reform has strengthened hospital discharge, there are different views on whether the discharge system has been made more effective. GPs are seen as responsible for coordination, but this does not reflect practice and is disputed by the pool of interviewees. As responsibilities of coordinating homecare, medical, and social services are separated, coordination depends on informal contacts between actors. Lack of financing for coordination tasks is considered as a major deficit. According to the Association of Swiss GPs and Pediatricians, coordination within LTC must be formally defined and linked to adequate financial rewards, contingent on meeting specific quality criteria (CH4-Mfe).

 Most Swiss organizations consider organizational fragmentation as the most challenging problem for coordination (CH6-Santésuisse). The Association of Social Service Organizations for the Elderly puts the main challenge at the fundamental governance barrier: the deep autonomy of cantonal and communal organizations prevents any top-down directive, requiring instead a slow, bottom-up consensus process for coordination (CH5-ProSenectute).

####  Local Government 

 In Switzerland, responsibility for community nursing and basic personal care has shifted to health insurance. Home-based LTC is usually provided by a public local nursing organization (Spitex), set as part of municipal healthcare and welfare services. This non-profit organization is mandated by municipalities as main actor in local LTC and social services. While public Spitex organizations primarily provide LTC, recipients may also choose services from private Spitex organizations that have a small market share.^65^ According to interviewees, the parallel system of public and private organizations poses substantial coordination problems. The interview addresses a two-tiered system: community-mandated, non-profit Spitex must deliver all care, including complex cases in remote areas, whereas private Spitex providers can skim less demanding patients and are free to “choose the cherries of the cake” (CH8-SSR).

####  Role of Professions

 Switzerland has no national gatekeeping and GPs coordinate LTC with a legal responsibility to assess elderly care. In practice, GPs do not act as coordinators for LTC. Interviewed organizations believe the current system works well and suggest improvements through more flexible funding for coordination tasks and service integration within local networks. Professional roles in coordination could be slightly adapted, but interviewees generally do not question the dominance of the medical profession (CH6-Santésuisse). Often LTC organizations make the decision on the level of care (CH8-SSR). Interviewees outline the formal process of the GP initiating a care order, a nurse then assesses on-site the needs and reports back. After that the GP gives the final approval to execute the care (CH2-BAG).

 GPs are seen as main actors for coordination due to their key role in care assessment, while in practice they rarely communicate with care providers and have limited insight into homecare and LTC. With few formal contact points between private Spitex organizations and GPs, a productive exchange depends on the voluntary initiative of individual professions (CH4-Mfe).

####  Digitalization

 In Switzerland, hospitals were required to introduce an electronic patient record system in 2020, while adoption remained voluntary for ambulatory services such as GP and Spitex-services, as well as for individual patients. The Swiss Hospital Organization calls the electronic patient record an ideal instrument for integrated networks but the “double voluntariness” results in a weak adoption mechanism (CH3-H+). Slow implementation is attributed to high costs and reluctance of older GPs to invest in electronic tools. The expectation is that patients will increasingly expect from GPs to participate, which is more likely by younger GPs (CH2-BAG).

###  Synthesis of Results

 Coordination is complicated by high levels of choice and competition, lower access regulation, and weaker positions of GPs and primary care centers. According to the institutional analysis, Sweden and the Netherlands have stronger healthcare and LTC links, and more homogenous systems. By contrast, in Germany and Switzerland, healthcare and LTC are more fragmented (See Table S1, Supplementary file 2).

 Interview partners in all four countries report a lack of cross-sectoral use of communication systems. Better integration of healthcare and LTC sectors also means better integration of their financing systems, with all countries emphasizing that DRG systems should have less influence on financial and personnel resources for care. According to the interviews, DRGs create incentives for one sector to refer patients to the other as quickly as possible. The more separate the two systems, the more likely it is that one sector shifts responsibilities to the other. In part as a response to consequences of DRG financing, special discharge teams organize transition processes to LTC.

 Within the primary healthcare sector, nurses and GPs tend to be overburdened with a heavy workload and lack of financing of coordination tasks, leaving little time for care coordination. In Sweden and the Netherlands, GPs act as gatekeepers. Here, GPs and primary care centers have a strong position and a coordinating role within the healthcare system that potentially can be extended to LTC. Interviewees in both countries are open to transferring responsibilities to allied health professionals, such as district nurses. In Switzerland, GPs are named key coordinators, though nurses make most decisions. In Germany, case managers like social workers are considered a solution to coordinate hospital and LTC. Coordination responsibilities in healthcare and LTC are bound by country-specific institutional and organizational frameworks. While Sweden and the Netherlands have built more structures to manage challenges, Germany and Switzerland leave more to individual navigation. Improving coordination requires tailoring strategies to each system’s institutional logic. In Sweden, formalization of county-employed GPs and municipality-employed district nurses could improve coordination, as professional silos and workplace pressures hinder integration. The Netherlands could empower municipalities to act as systems integrators for its complex public-private landscape and to help oversee and navigate its competitive market. For Germany, a cross-sectoral case manager role to navigate deeply fragmented systems could help. Here, the core challenge is the lack of existing actors responsible for coordination as well as early stages of digitalization of data storing and sharing. Switzerland could focus on formalizing its effective but informal local network, as a main challenge is its decentralized structure.

 Still, common themes that hinder seamless care across all four advanced welfare states remain. Underlying issues exist even under differing institutional logics. Inadequate funding for coordination, misaligned financial incentives, communication barriers, workforce pressures, and digital divides are central issues in all countries. From the analysis of the interviews, general recommendations to improve coordination emerged.

 Sometimes, separation of healthcare and LTC budgets leads to conflicts and negotiations between counties and municipalities about responsibilities for certain services. To reduce intersectoral divide, organizations suggest that instead of top-down coordination, flexible, local networks should assure better coordination of services. Organizations criticize that care coordination is an unpaid activity in addition to other tasks that should become visible in budgets. Interviewed organizations suggest empowering local government by having hospitals recommend an initial needs assessment for the first days following discharge. Once patients have adjusted to homecare, the municipality would perform the final assessment. Preliminary recommendations by the hospital would speed up the discharge process yet municipalities need to build up trust in hospitals’ recommendations. Organizations emphasize the need for a single coordinator who can be directly contacted by patients and their families. A solution is seen in establishing homecare physicians with coordination responsibilities or independent case managers who accompany transfer between sectors. After discharge, organizations demand more direct communication between GPs and LTC nurses. This is challenging due to time and financial constraints, as well as differing competences between professions.

## Discussion

 Hospital care is medically dominated, case-oriented, and often economically influenced by DRGs. Patients are often discharged once acute medical treatment ends before all care needs are managed or they can independently handle daily activities, raising early readmission risk. Integrating medical, social and other care services into a house setting remains challenging. Effective coordination is needed, whether through professional or organizational dominance (such as one profession leading coordination, like a GP or homecare providers) or through collaboration and joint management.

 Germany and Switzerland face system fragmentation creating coordination challenges outside hospitals. Germany, with fragmented financing of hospital and LTC, heavily relies on family members for coordinating, providing, and financing care. In Switzerland, separation of responsibilities among various actors leads to informal coordination and its success depends on navigation abilities of single actors. The Netherlands and Sweden have advanced system integration in healthcare and LTC. The Netherlands has integrated hospital, outpatient, and homecare. Sweden connects primary, secondary, and LTC despite institutional separations, and offers financial incentives to prioritize homecare over institutional care.

 GPs and primary care centers play a central role in care coordination as the first point of contact. Gatekeeping by GPs exists in the Netherlands and Sweden, although Sweden lacks a strong regulatory framework for gatekeeping. In Switzerland, GPs are seen as potential coordinators but they do not see themselves as such, holding a legal responsibility for assessing, not for coordinating elderly care. In Germany, GPs are not gatekeepers or coordinators, though they could assume coordination responsibilities. Organizations in all four countries report that GPs are being overworked. Allied health professionals participate in care coordination to varying degrees in the four countries, mostly from primary care side or inpatient discharge planning. Many organizations highlight a lack of formal funding for care coordination.

 Interviews in all countries show the importance of clear responsibilities to better connect healthcare and LTC. In Sweden and the Netherlands, where municipalities oversee LTC, homecare has been strengthened and access to LTC homes has become more restricted. Coordinating services is essential in homecare, involving various healthcare and social care providers. The Swiss case demonstrates that a public non-governmental organization (Spitex), alongside municipalities, could also take on responsibility for LTC, improving cooperation with hospitals during the discharge process. Assigning a responsible actor (municipalities in Sweden and the Netherlands, Spitex in Switzerland) greatly relieves patients and their families. In systems with competing actors, as in Germany, care coordination is reduced. In Switzerland and the Netherlands, competition among independent providers is seen as problematic, increasing coordination demands and allowing profit-oriented care organizations to “cherry pick” (CH8-SSR). In Sweden, municipalities lead primary healthcare and LTC, with district nurses playing a key coordinating role, especially in collaboration with homecare providers.

 Digitalization is considered not as replacing coordinating actors but as a support tool. In Germany and Switzerland, where GPs have no gatekeeping role and where primary healthcare centers are less developed, older GPs are unwilling to invest in digitalization. In Sweden and the Netherlands, where GPs coordinate primary healthcare and referrals to specialists, interviewees expressed strong interest in digitalization to improve communication and coordination. Still, digital systems for healthcare and LTC are not fully harmonized. First model projects suggest that integrating electronic records across sectors may facilitate coordination across healthcare and LTC. This analysis suggests a tempered enthusiasm for digitalization: while it holds immense promise, a blind faith in technology is naive. Digital tools do not replace the need for clear professional responsibilities and well-designed care pathways; rather, interviewees described their usefulness as dependent on how well they are integrated into existing structural frameworks.

 The comparative analysis demonstrates that, despite different institutional logics, the goal of care coordination remains universal. More effective care coordination ensures timely transitions, prevents avoidable readmissions, fulfills patient and family expectations, and provides value for public expenditure. While some countries in this study are leading in digitalization and integrated care coordination, their advanced experiences offer valuable insights into potential future pathways for other nations developing their own systems.

 This paper also extends and triangulates the quantifying typologies with qualitative data. While the typologies describe the institutional logics, qualitative data can trace the mechanisms and challenges of that logic for organizations and in everyday practice, such as varying degrees of incentives, coordination culture, and trust. The paper suggests that the friction between these two nested institutional logics of healthcare and LTC systems within a single country might be a primary driver of coordination failure. A public-public system such as Sweden can be informally disjointed, while a private-private system such as Switzerland can be informally cohesive. This suggests that while the typology establishes the need for coordination, the informal professional culture governs its solutions.

###  Limitations

 Two aspects potentially limit comparability: language nuances in non-native English interviews and the timing of interviews relative to the COVID-19 pandemic. Post-pandemic onset interviews included questions on its impact, though cross-country comparisons on this topic were not detailed due to varying data collection times. Another limitation is that the sample represents four of five healthcare and three of six LTC types. Therefore, our findings cannot claim full typological coverage. Yet, the included combinations cover both highly regulated and market-oriented systems, providing a robust test of coordination mechanisms across institutional diversity. Future research could examine remaining types to test whether observed coordination logics extend to, for example, residual or Southern European models. This study does not compare typologies in relation to outcomes, such as waiting times for LTC, cost-effectiveness of care pathways, or patient and caregiver satisfaction scores.

## Disclosure of artificial intelligence (AI) use

 Not applicable.

## Ethical issues

 No ethical issues to be revealed. For the article we reanalysed interviews from a German Research Association (DFG) funded research project “Comparing the coordination of elderly care services in European welfare states: how organizational actors respond to marketization policies.” Interview data were stored on the legal basis of the EU General Data Protection Regulation (GDPR) in accordance with Art. 6 Par. 1 DSGVO (German data protection law). For this research project (expert interviews) no ethical approval was required nor for the reuse of already collected interview data.

## Conflicts of interest

 Authors declare that they have no conflicts of interest.

## Disclaimer

 This paper is written by the authors, and is the product of professional research.

## Supplementary files


Supplementary file 1. List of Organizations.

Supplementary file 2 contains Table S1.
