# Supplementary material for: Coordinating Healthcare and Long-term Care: A Four-Country Comparison
Source: Int J Health Policy Manag. 2026 Jul 12;15:9130. doi: 10.34172/ijhpm.9130 (PMC13770234; doi:10.34172/ijhpm.9130)
Supplement: Supplementary file 1 [file ijhpm-15-9130-s001.pdf]

Article title: Coordinating Healthcare and Long-term Care: A Four-Country Comparison

Journal name: International Journal of Health Policy and Management (IJHPM)

Authors' information: Claus Wendt[1]*, Eva Willis[2]

[1]Chair of Sociology of Health and Healthcare Systems, University of Siegen, Siegen, Germany.

[2]Sociology of Health and Healthcare Systems, University of Siegen, Siegen, Germany.

***Correspondence to:** Claus Wendt; Email: wendt@soziologie.uni-siegen.de

Citation: Wendt C, Willis E. Coordinating healthcare and long-term care: a four-country comparison. Int J Health Policy Manag. 2026;15:9130. doi:10.34172/ijhpm.9130

Supplementary file 1. List of Organizations

Germany					
Abbreviation	Full national name	English translation	Main task*	Date	Type of survey
BAGFW	Bundesarbeitsgemeinschaft der Freien Wohlfahrtspflege e. V.	Federal Association of Non-statutory Welfare	(Home) care provider	21.09.2020	Online
BAGSO	Bundesarbeitsgemeinschaft der Seniorenorganisationen	German National Association of Senior Citizens' Organisations	Other	09.10.2020	Online
Bpa	Bundesverband privater Anbieter sozialer Dienste e.V.	Federal Association of Private Providers of Social Services	Home care provider	14.02.2020	Face to face
BV Geriatrie	Bundesverband Geriatrie	German Federal Geriatrics Association	Geriatrician	26.11.2020	Online
Care support center	Pflegestützpunkt der Caritas	Care support center	Home care provider	10.09.2019	Face to face
DAlzG	Deutsche Alzheimer Gesellschaft e.V. Selbsthilfe Demenz	German Alzheimer Association	Other	11.09.2020	Online
Discharge management	Entlassmanagement einer deutschen Universitätsklinik	Discharge management of a university hospital	transfer nurses	10.09.2019	Face to face

DKG	Deutsche Krankenhausgesellschaft	German Hospital Federation	Hospital	05.10.2020	Online
DPR	Deutscher Pflegerat e.V.	German Nursing Council	LTC nurses	19.02.2020	Face to face
DPR	Deutscher Pflegerat e.V.	German Nursing Council	LTC nurses	07.10.2020	Online
DV	Deutscher Verein für öffentliche und private Fürsorge e. V.	German Association for Public and Private Welfare	Other	21.09.2020	Online
DVSG	Deutsche Vereinigung für Soziale Arbeit im Gesundheitswesen e. V.	German Association for Social Work in Health Care	Other	20.10.2020	Online
GKV	GKV Spitzenverband	National Association of Statutory Health Insurance Funds	Funding agency	23.10.2020	Online

HÄV	Deutscher Hausärzteverband	German Association of Family Doctors	GP	13.10.2020	Online
KBV	Kassenärztliche Bundesvereinigung	The National Association of Statutory Health Insurance Physicians	GP	30.11.2020	Online
KDA	Kuratorium deutsche Altershilfe	Board of Trustees for German Old Age Assistance	Other	12.11.2020	Online
MD	Medizinischer Dienst	Medical Service	Care assessment	22.10.2020	Online
Netherlands					
Abbreviation	Full national name	English translation	Main task	Date	Type of survey
Actiz	ActiZ	Organization of employers of care facilities	Home care provider	19.11.2019	Face to face
CIZ	Centrum Indicatiestelling Zorg	Needs asessment center	Care assessment	08.10.2019	Face to face
Geriatrician	[individual/ non-organizaional actor]	Geriatrician working in a university hospital	Geriatrician	14.01.2020	Face to face
Home care nurse	[individual/ non-organizational actor)	Home care nurse	LTC nurse	18.10.2019	Face to face
Huisarts	[individual/ non-organizational actor)	GP	GP	29.10.2019	Face to face
Ineen	InEen	Association of primary care providers	GP	16.10.2019	Face to face
NHG	Nederlandse Huisartsen Genootschap	Dutch General Practitioners Association	GP	16.01.2020	Face to face
NZa	Nederlandse Zorgautoriteit	Dutch Healthcare Authority	Supervisory agency	22.10.2019	Face to face
Patientenfederatie	Patiëntenfederatie Nederland	Dutch patient organization	Other	24.10.2019	Face to face
Transfer nurse	[individual/ non-organizational actor]	Transfer nurse	Transfer nurse	22.11.2019	Face to face

Ucentraal	Ucentraal	Welfare organization with a focus on organizing voluntary work	Other	07.11.2019	Face to face
V&VN	Beroepsvereniging Verzorgenden Verpleegkundigen	National Association of Nurses and Carers in the Netherlands	LTC nurse	11.11.2019	Face to face
Verenso	Vereniging van specialisten ouderengeneeskunde	Dutch Association of Elderly Care Physicians	Geriatrician	12.11.2019	Face to face
VGZ	VGZ Zoorgkantor	Care Office	Funding agency	03.12.2019	Face to face
ZN	Zorgverzekeraars Nederland	Umbrella organization of health insurers	Funding agency	14.11.2019	Face to face
Sweden					
Abbreviation	Full national name	English translation	Main task	Date	Type of survey
Äldreomsorg	Äldreomsorg [pseudonymized a sit is a small organization]	Municipal public care provider	Home care provider	13.02.2020	Face to face
Famna	Riksorganisationen för idéburen vård och social omsorg	Association of non-profit care providers	(Home) care provider	04.09.2020	Online
IVO	Inspektionen för vård och omsorg	Health and Social Care Inspectorate	Supervisory agency	26.11.2020	Online
Qultrum	Qultrum	Center for Learning and Innovation in Healthcare	(LTC) nurse	17.09.2020	Online
Regional council	Region Stockholm	Regional council	Healthcare provider	06.02.2020	Face to face
SBU	Statens beredning för medicinisk och social utvärdering	Swedish Agency for Health Technology Assessment and Assessment of Social Services	Supervisory agency	03.02.2020	Face to face
Socialstyrelsen	Socialstyrelsen	National Board of Health and Welfare	Supervisory agency	31.01.2020	Face to face
SSF	Svensk sjuksköterskeförening	Swedish Society of Nursing	(LTC/District) Nurse	23.09.2020	Online
Tiohundra	Tiohundra AB	Provider of integrated care	Healthcare and home care provider	01.09.2020	Online

Tryggt mottagande	Tryggt mottagande [pseudonymized as it is a small organization]	Municipal hospital discharge management	Transfer nurses	18.09.2020	Online
Switzerland					
Abbreviation	Full national name	English translation	Main task	Date	Type of survey
ASPS	Verband der privaten Spitex-Organisationen<br>Association Spitex privée Suisse	Association of private basic care organizations	(Home)care provider	04.03.2020	Face to face
BAG	Bundesamt für Gesundheit<br>Office fédéral de la santé publique	Federal Office of Public Health (FOPH)	Supervisory agency	05.03.2020	Face to face
H+	Die Spitäler der Schweiz<br>Les Hôpitaux de Suisse	Swiss Hospital Organization	Hospital	06.03.2020	Face to face
Mfe	Haus- und Kinderärzte Schweiz<br>Médecins de famille et de l'enfance Suisse	Association of Swiss GPs and Pediatricians	GP	14.02.2020	Face to face
Pro Senectute	Soziale Dienste für ältere Menschen	Association of social services organizations for the elderly	(Home) care provider	12.03.2020	Face to face
Santésuisse	Branchenorganisation der Schweizer Krankenversicherer Santésuisse	Association of Swiss Health Insurance Organizations	Funding agency	12.03.2020	Face to face
SBK	Schweizer Berufsverband der Pflegefachfrauen und Pflegefachmänner<br>Association suisse des infirmières et infirmiers	Swiss Association of Occupational Carers	LTC nurse	05.03.2022	Face to face
SSR	Schweizerischer Seniorenrat<br>Conseil suisse des aînés	Swiss organization of elderly persons	Other	11.03.2020	Face to face

Source: Bahle et al. 2023, 155-159

*As some organizations fulfill several functions and cover multiple areas, this section names the main task of each organization referring to the focus of the interviews
