# Supplementary material for: Coordinating Healthcare and Long-term Care: A Four-Country Comparison
Source: Int J Health Policy Manag. 2026 Jul 12;15:9130. doi: 10.34172/ijhpm.9130 (PMC13770234; doi:10.34172/ijhpm.9130)
Supplement: Supplementary file 2 [file ijhpm-15-9130-s002.pdf]

Article title: Coordinating Healthcare and Long-term Care: A Four-Country Comparison

Journal name: International Journal of Health Policy and Management (IJHPM)

Authors' information: Claus Wendt[1]*, Eva Willis[2]

[1]Chair of Sociology of Health and Healthcare Systems, University of Siegen, Siegen, Germany.

[2]Sociology of Health and Healthcare Systems, University of Siegen, Siegen, Germany.

***Correspondence to:** Claus Wendt; Email: wendt@soziologie.uni-siegen.de

Citation: Wendt C, Willis E. Coordinating healthcare and long-term care: a four-country comparison. Int J Health Policy Manag. 2026;15:9130. doi:10.34172/ijhpm.9130

Supplementary file 2

Supplementary Material

Comparative synthesis of 4 country healthcare and long-term care systems

Category	Sweden (Public HC - Public LTC)	Netherlands (Regulation-oriented Public HC - Need-based private LTC)	Germany (Supply/Choice-oriented Public HC - Private Supply LTC)	Switzerland (Supply-oriented Private HC - Need-based Private LTC)
**1. Division of HC G LTC Sectors**	**Institutionally separated but linked.** Hospitals (county responsibility) and LTC (municipal responsibility) are separate, but linked by strong financial incentives (e.g., municipalities pay for delayed discharges) and laws to enforce collaboration.	**Integrated yet complex.** Hospital, outpatient, and homecare are more integrated, with health insurance now financing homecare. Responsibilities are split between national, regional, and municipal levels, leading to cost-shifting.	**Strict fragmentation.** HC and LTC are strictly separated in financing and organization. HC is further divided into inpatient and outpatient sectors. This creates major discontinuities.	**High fragmentation with separated responsibilities.** HC is private and LTC is need-based. Responsibilities for coordinating homecare medical, and social services are separated among many actors, relying on informal contacts.
**2. Local Government**	**Strong financial incentive to coordinate.** Municipalities are financially responsible for LTC and must compensate hospitals for delayed discharges, creating a powerful incentive to organize efficient homecare.	**Major actor with financial power.** Since the 2015 reform, municipalities are responsible for social care and non-residential LTC. Municipalities have significant influence and financial allocation powers.	**Limited role.** Municipalities are hardly involved in HC or homecare, acting only as a last safety net. They are not seen as central coordinators.	**Delegated authority.** Municipalities mandate public non-profit Spitex organizations as the main local LTC actors. Coordination is challenged by a parallel system of private providers.
**3. Role of Professions**	**GPs G District Nurses as key, but under pressure.** GPs and primary care centers coordinate within HC. District nurses (county or municipal) are pivotal coordinators. Collaboration is crucial but strained by workload.	**GPs as gatekeepers, but shared responsibility.** GPs have a strong gatekeeping role but are overworked. Coordination is increasingly shared with community nurses, transfer nurses, and geriatricians. Nursing academization is high.	**No formal coordination role for GPs.** No gatekeepers, overburdened. Coordination often falls to family members or is suggested for independent case managers/social workers. Nursing academization is low.	**GPs legally responsible but not practicing coordinators.** GPs hold legal responsibility for care assessment but rarely communicate with LTC providers. In practice, nurses in Spitex organizations make most coordination decisions.
**4. Digitalization**	**Advanced but sectorally divided.** Digitalization is a national priority with advanced eHealth. A major deficit is the separate electronic system for LTC, which denies staff access to the central medical database.	**Leader with incompatible systems.** A leader in digitalization with widespread use of electronic systems. However, systems between HC and LTC are not compatible, hindering information exchange.	**Early stage with significant barriers.** Electronic records are not fully implemented and are limited to HC. Uptake is hindered by high costs, older GPs, and data protection concerns.	**Slow implementation due to 'double voluntariness'.** Adoption of electronic patient records is mandatory for hospitals but voluntary for GPs and LTC providers. This, and reluctance from older GPs, slows progress.
